# Antibody–drug conjugates in gastric cancer: from targeted cytotoxicity to immune modulation

**DOI:** 10.3389/fimmu.2026.1928576

**Published:** 2026-09-23

**Authors:** Honghua Su, Chunyin Yang, Jiayun Ge

**Affiliations:** Department of General Surgery, Kunming Municipal Hospital of Traditional Chinese Medicine, The Third Affiliated Hospital of Yunnan University of Chinese Medicine, Kunming, Yunnan, China

**Keywords:** antibody–drug conjugates, Claudin 18.2, drug resistance, gastric cancer, HER2, immunotherapy, nectin-4, targeted therapy

## Abstract

Gastric cancer (GC) remains a major cause of cancer-related mortality, and therapeutic resistance and tumor heterogeneity continue to limit durable responses in advanced disease. Antibody–drug conjugates (ADCs), which combine the target specificity of monoclonal antibodies with highly potent cytotoxic payloads, have emerged as an important component of biomarker-guided therapy. Beyond selective payload delivery, accumulating evidence indicates that ADCs can interact with antitumor immunity through Fc-mediated immune-effector functions, immunogenic tumor-cell death, enhanced antigen presentation, recruitment of effector T cells, and remodeling of the tumor immune microenvironment. In this review, we summarize the structural and pharmacological principles of ADCs and discuss established and emerging targets in GC, including HER2, CLDN18.2, HER3, GCC, TROP2, Nectin-4, EGFR, and MUC1. Particular emphasis is placed on the immunological consequences of ADC treatment and the mechanistic rationale for combining ADCs with immune checkpoint inhibitors. We further discuss tumor heterogeneity, resistance mechanisms, biomarker selection, treatment-related toxicities, and emerging combination strategies. Integrating tumor-antigen biology with immune-contextual features may enable more rational patient selection and therapeutic combinations. Collectively, next-generation ADCs may function not only as targeted cytotoxic agents but also as modulators of antitumor immunity in gastric cancer.

## Introduction

Gastric cancer(GC) is the fifth most prevalent cancer globally in terms of both incidence and mortality, with nearly 1 million new diagnoses each year and over 650,000 deaths worldwide, according to the GLOBOCAN 2022 data (1). It is a heterogeneous disease, with two primary histological subtypes identified by the Laurén classification: intestinal and diffuse types. Intestinal type GC is associated with H. pylori infection, while diffuse type GC is characterized by poorly cohesive cells and signet ring cells, often resulting in poor prognosis and resistance to systemic therapy (2). The Cancer Genome Atlas identified distinct molecular subtypes of GC, including chromosomal instability (CIN), microsatellite instability (MSI), genome stability, and Epstein-Barr virus (EBV) positivity, each associated with specific genetic and immunological features that influence prognosis and therapy response. Environmental factors, including H. pylori infection, high salt intake, obesity, and gastroesophageal reflux, play a significant role in GC pathogenesis, with familial aggregation observed in about 10% of cases (**Figure 1**). Known heritable mutations, including those in CDH1 and genes encoding homologous recombination deficiency, contribute to the hereditary forms of GC (3–6). Despite the diverse genomic landscape, the Laurén classification remains widely used in clinical practice, though the clinical relevance of certain molecular subgroups remains unclear.

**Figure 1:**
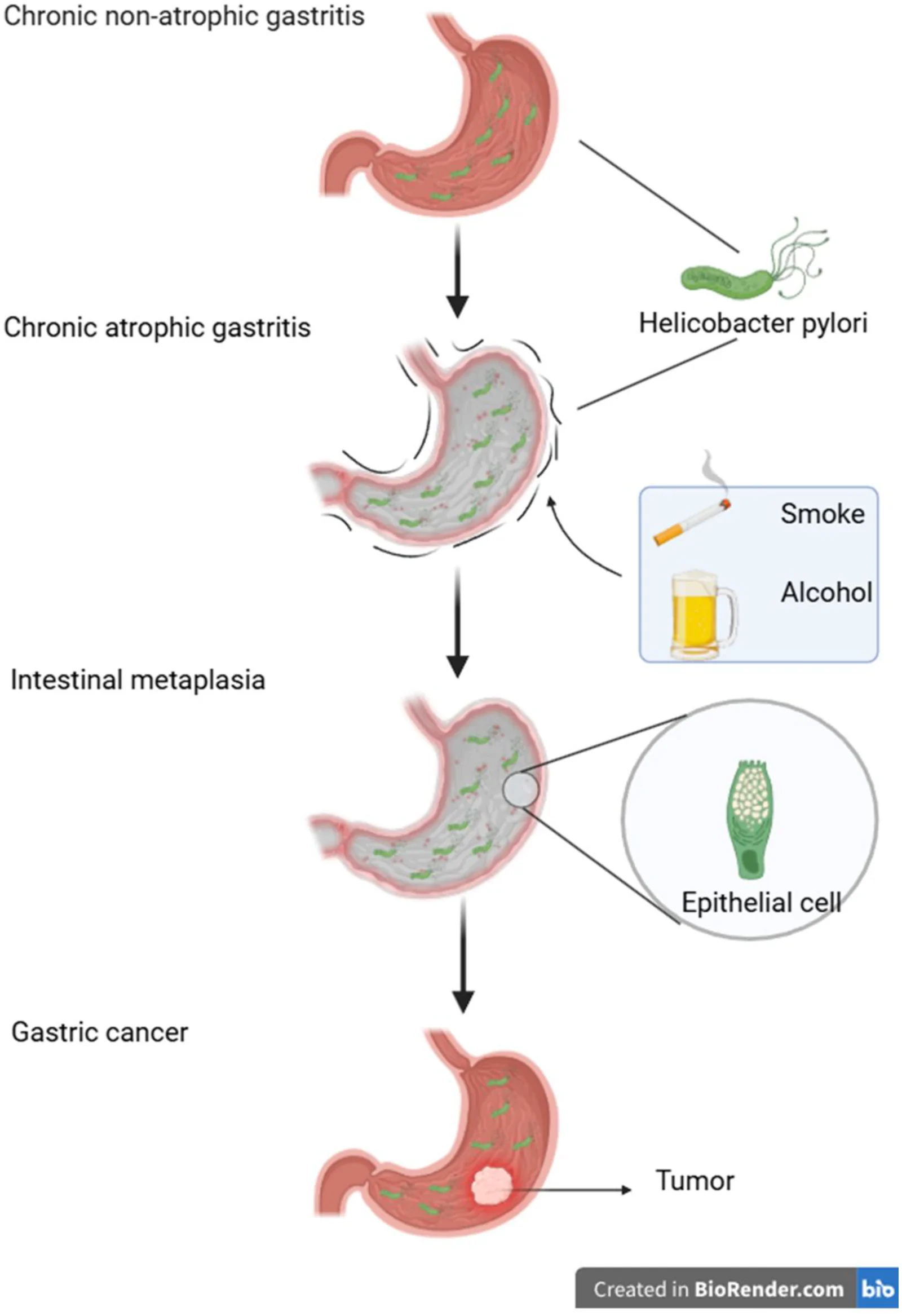
Evolution of GC and its causes. Created with BioRender.com.

Most GC patients are diagnosed at advanced stages, which correlates with poor prognosis (7). Surgical intervention remains effective for early-stage gastric cancer, whereas advanced disease generally requires multimodal treatment, including surgery, chemotherapy, radiotherapy, targeted therapy, and immunotherapy (8). For patients with HER2-positive advanced gastric or gastroesophageal junction adenocarcinoma, trastuzumab-based therapy is an established component of first-line treatment. More recently, the addition of pembrolizumab to trastuzumab plus fluoropyrimidine- and platinum-based chemotherapy has further expanded first-line treatment for patients with PD-L1 CPS ≥1 (9). However, most GC patients are diagnosed at later stages, limiting curative options due to the asymptomatic nature of the disease (10, 11). Thus, gaining a deeper understanding of the molecular mechanisms involved in GC progression and metastasis is crucial for discovering new therapeutic targets that could improve patient outcomes. For patients with intermediate and advanced GC, targeted therapies based on specific molecular targets have marked a significant advancement in precision medicine (12). Targeted therapies, developed to interact with defined molecular sites within tumor cells, offer selective destruction of tumor cells while minimizing harm to healthy tissue (13, 14). Despite their benefits, targeted therapies are not without challenges, including adverse effects and the potential for resistance. Resistance can either be primary, where no therapeutic response occurs initially, or secondary, where an initially effective treatment loses its efficacy over time (15). As a result, identifying novel therapeutic strategies and targets is crucial to overcome drug resistance and enhance clinical outcomes (15, 16).

Advances in antibody engineering, linker chemistry, payload design, and conjugation strategies have transformed ADCs into a clinically validated therapeutic platform across hematological and solid malignancies (17–19).

## Mechanism of ADC action and design determinants

Antibody–drug conjugates are modular therapeutics composed of a tumor-targeting monoclonal antibody, a linker, and a cytotoxic payload. Following binding to a cell-surface antigen, the ADC–antigen complex is internalized and trafficked through the endosomal–lysosomal pathway, where linker cleavage and/or antibody degradation releases the active payload. The released drug induces tumor-cell death through mechanisms such as microtubule disruption or DNA/topoisomerase damage, while membrane-permeable payloads may diffuse into neighboring antigen-low cells and generate a bystander effect (20–23). Depending on Fc engineering, the antibody component may additionally retain immune-effector activity (22, 23). These core steps in ADC structure, intracellular trafficking, payload release, direct cytotoxicity, and bystander killing are summarized schematically in **Figure 2**.

**Figure 2:**
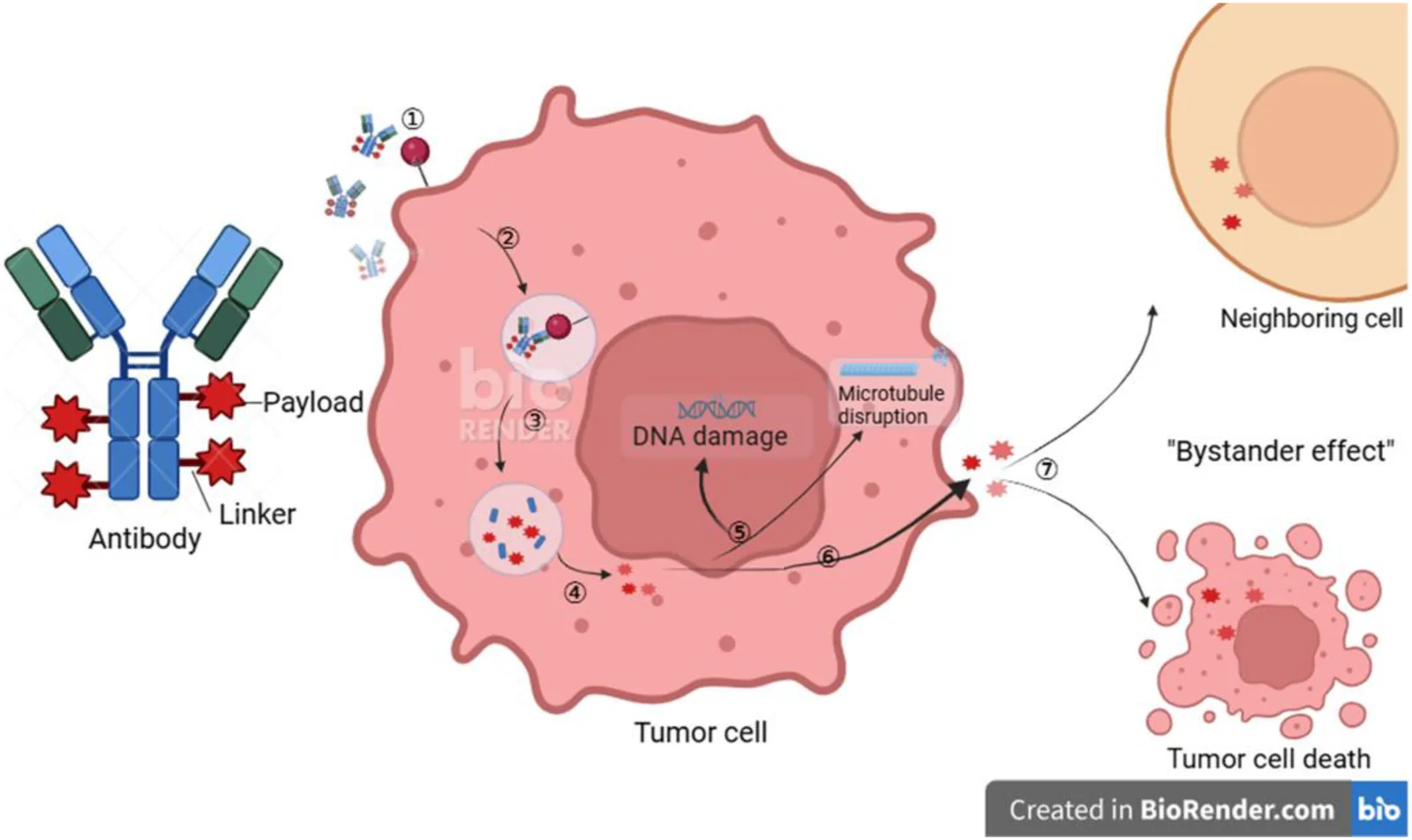
Core mechanisms of antibody–drug conjugate action. Created with BioRender.com.

Rather than acting independently, the antibody, linker, payload, conjugation characteristics, and drug-to-antibody ratio (DAR) collectively determine ADC efficacy, pharmacokinetics, and safety (18, 19). DAR influences payload loading and can also affect physicochemical properties, systemic exposure, and clearance; however, ADC pharmacokinetics are additionally influenced by antibody properties, target-mediated disposition, linker stability and deconjugation, conjugation characteristics, and payload properties (18, 19, 24). Linker chemistry is particularly important because it governs plasma stability, intracellular payload-release kinetics, and payload diffusibility, thereby influencing pharmacokinetics, bystander activity, therapeutic index, and off-target toxicity. Recent advances in linker design, including enzyme-responsive and tumor-selective cleavage mechanisms, hydrophilicity-modulating spacers, self-immolative structures, and other optimized release strategies, aim to improve systemic stability while maintaining efficient payload release within tumor cells (25, 26). Antitumor activity further depends on antigen expression and heterogeneity, internalization efficiency, tumor penetration, payload sensitivity, and bystander activity (19, 24, 27).

## Immunological mechanisms of ADCs in gastric cancer

Beyond their canonical function as tumor-targeted cytotoxic delivery systems, ADCs can interact with antitumor immunity through both their antibody and payload components. Depending on Fc engineering, the antibody component may retain immune-effector activity, while IgG1-based ADCs can engage Fcγ receptors and contribute to antibody-dependent cellular cytotoxicity(ADCC) and complement-dependent cytotoxicity (19, 22, 23, 28). ADC-associated tumor-cell killing may also induce immunogenic cell death, which can promote dendritic-cell activation, antigen presentation, and subsequent effector T-cell responses (29, 30).

Preclinical evidence further supports direct immunomodulatory effects of HER2-targeted ADCs. Trastuzumab deruxtecan (T-DXd; formerly DS-8201a) has been shown to increase tumor-infiltrating dendritic cells and CD8^+^ T cells and to upregulate MHC class I and PD-L1 expression, indicating that ADC-mediated cytotoxicity may be accompanied by remodeling of antitumor immunity (31). Such immune activation may simultaneously enhance tumor immunogenicity and induce compensatory checkpoint signaling, providing a mechanistic rationale for combining ADCs with PD-1/PD-L1 blockade. Consistent with this concept, synergistic antitumor activity between HER2-directed ADCs and checkpoint blockade has been demonstrated in preclinical models (31, 32).

## ADC targets and clinical development in gastric cancer

ADC development in gastric cancer has expanded from HER2-centered strategies to a broader spectrum of tumor-associated antigens. The following section summarizes major ADC targets according to their biological relevance, clinical maturity, and therapeutic potential. Representative ADCs and their key structural and clinical characteristics are summarized in **Table 1**.

**Table 1:** Composition of antibody–drug conjugates for gastric cancer.

NCI thesaurus code	ADC name	Target	Antibody	Payload	Linker	DAR
C128799	Trastuzumab deruxtecan(T-DXd)	HER2	Trastuzumab	DXd	Tetrapeptide cleavable	~8
C169918	Disitamab vedotin (RC48)	HER2	Disitamab	MMAE	vc-PABC	~4
N/A	Sacituzumab govitecan-hziy	TROP2	hRS7	SN-38	Cleavable	~7.6
N/A	Sacituzumab tirumotecan	TROP2	anti-TROP2	Undisclosed	Cleavable	N/A
N/A	YL-242	CLDN18.2	anti-CLDN18.2	Topo I inhibitor	Cleavable	—
N/A	7MW-4911	CDH17	anti-CDH17	Topo I inhibitor	Cleavable	—

The development of ADC targets in gastric cancer has evolved from an initially HER2-centered paradigm toward progressive diversification of the actionable antigen landscape. Early clinical development was dominated by HER2-directed ADCs, whereas subsequent efforts expanded toward gastric-lineage and epithelial targets such as CLDN18.2, HER3, GCC, TROP2, Nectin-4, EGFR, and MUC1. More recently, emerging strategies have begun to explore immune-associated surface antigens, including B7-H3, as well as additional lineage-restricted targets such as CDH17. This evolution reflects a broader shift from targeting a single dominant oncogenic receptor toward exploiting tumor lineage, immune-regulatory biology, antigen heterogeneity, and treatment-induced target plasticity. The evolution and current developmental status of representative ADC targets in gastric cancer are summarized in **Table 2**.

**Table 2:** Evolution and current landscape of ADC targets in gastric cancer.

Development wave	Target	Representative ADCs	Biological rationale in GC	Current status/relevance
Established HER2-centered era	HER2	T-DM1, T-DXd, RC48	Clinically validated oncogenic target; immune and molecular heterogeneity	Most mature GC ADC target; T-DXd and RC48 provide clinical proof of concept
Target-diversification era	CLDN18.2	CMG901/AZD0901, LM-302, ATG-022	Gastric-lineage restricted antigen	Rapid clinical development; among the most promising emerging GC targets
Target-diversification era	HER3	HER3-DXd/U3-1402	HER-family signaling; associated with adverse prognosis	Early/preclinical development in GC
Target-diversification era	TROP2	Sacituzumab govitecan, sacituzumab tirumotecan	Broad epithelial expression; efficient internalization	Clinical evidence in GC remains limited
Target-diversification era	GCC	TAK-164, TAK-264	Gastrointestinal lineage-associated receptor	Clinical development limited by efficacy/therapeutic index
Target-diversification era	Nectin-4	Nectin-4-directed ADCs	Associated with progression; potential target after HER2 loss	Emerging GC target; relevant to target switching
Target-diversification era	EGFR	MRG003	Commonly dysregulated receptor	Biomarker refinement required
Target-diversification era	MUC1	DS-3939a and other MUC1 ADCs	Tumor-associated glycosylation	Early clinical/preclinical development
Emerging immune-associated targets	B7-H3 (CD276)	YL201, I-DXd	Immune-regulatory molecule; associated with aggressive biology and immune escape	Early pan-tumor clinical development; GC-specific efficacy not yet established
Emerging lineage targets	CDH17	7MW-4911	Gastrointestinal lineage-associated surface antigen	Emerging/preclinical or early clinical development

### HER2

HER2 is the most clinically established ADC target in gastric cancer. Beyond its canonical role in promoting tumor-cell proliferation and survival, HER2-positive gastric cancer is also characterized by distinct immune features. HER2-associated molecular alterations have been linked to remodeling of the tumor immune microenvironment and to PD-L1 expression, suggesting that HER2 biology may influence both tumor-intrinsic signaling and antitumor immunity (33, 34). These biological and immunological features provide a rationale for HER2-directed ADCs and for therapeutic strategies integrating HER2 targeting with immune modulation.

The clinical evolution of HER2-directed ADCs illustrates that target expression alone is insufficient to determine therapeutic efficacy. Although trastuzumab emtansine (T-DM1) showed potent antitumor activity in preclinical models of HER2-positive gastric cancer (35), the phase II/III GATSBY trial failed to demonstrate a survival advantage over taxane chemotherapy (36). Several factors may explain this negative result. Gastric cancer is characterized by pronounced spatial and intratumoral heterogeneity of HER2 expression, and biomarker analysis of GATSBY indicated that T-DM1 activity was greater in tumors with higher HER2 expression, suggesting that antigen heterogeneity and insufficient target density may limit effective payload delivery (37). In addition, T-DM1 uses a non-cleavable linker and releases a poorly membrane-permeable DM1-containing catabolite after lysosomal degradation, resulting in limited bystander killing of adjacent HER2-low or HER2-negative tumor cells. This feature may be particularly disadvantageous in heterogeneous gastric tumors. In contrast, T-DXd combines a higher drug-to-antibody ratio with a cleavable linker and a membrane-permeable topoisomerase I inhibitor payload, enabling a more pronounced bystander effect (38). These pharmacological differences may partly explain why T-DXd subsequently achieved substantial improvements in tumor response and survival in the DESTINY-Gastric01 trial (39). Disitamab vedotin (RC48) has also shown clinically meaningful activity in advanced HER2-expressing gastric or gastroesophageal junction cancer (40). Collectively, these contrasting outcomes indicate that the efficacy of HER2-directed ADCs is determined not only by HER2 positivity, but also by antigen density and heterogeneity, ADC architecture, payload properties, and bystander activity.

Next-generation HER2-directed ADCs are being developed to address persistent challenges such as heterogeneous target expression and acquired resistance. ARX788 has demonstrated encouraging activity in HER2-positive advanced gastric and gastroesophageal junction adenocarcinoma in early-phase clinical evaluation (41), whereas MRG002 and XMT-1522 have shown potent preclinical activity in HER2-expressing tumors, including models with resistance to earlier HER2-directed therapies (42, 43). Rather than simply expanding the number of HER2-targeted agents, these approaches reflect a broader shift toward optimizing conjugation strategies, payload delivery, bystander activity, and activity in treatment-resistant disease. Thus, the development of HER2-directed ADCs provides a paradigm for how target biology and ADC design must be considered together to overcome heterogeneity and resistance in gastric cancer.

### HER3

HER3 expression has been associated with poorer survival in GC (44). HER3-directed ADCs remain exploratory; importantly, HER3-DXd has shown the ability to induce immune activation and sensitize tumors to PD-1 blockade (45), while EV20/NMS-P945 provides additional preclinical evidence supporting HER3-directed ADC development (46).

### CLDN18.2

CLDN18.2 has emerged as one of the most promising gastric-lineage targets because its membrane accessibility increases following malignant transformation while expression is retained in a substantial subset of gastric cancers (47, 48). Although zolbetuximab is a CLDN18.2-directed monoclonal antibody rather than an ADC, its clinical success has validated CLDN18.2 as a therapeutically actionable target in gastric and gastroesophageal junction cancer (49). This clinical validation has provided a strong rationale for the subsequent development of CLDN18.2-directed ADCs, including CMG901 and other emerging agents.

Among these agents, CMG901 (AZD0901) has generated the most mature clinical evidence. In the KYM901 phase 1 study, CMG901 demonstrated encouraging antitumor activity in patients with advanced CLDN18.2-positive gastric or gastroesophageal junction cancer, supporting further development of this ADC platform (50, 51). Other agents, including SYSA1801, LM-302, ATG-022, and related CLDN18.2-directed ADCs, remain in early clinical or preclinical development (52–54). Collectively, CLDN18.2 provides an important example of how lineage-restricted antigen expression can be translated into ADC development; however, optimal expression thresholds, spatial heterogeneity, and mechanisms of acquired resistance remain important questions for future investigation.

### GCC

Guanylate cyclase C (GCC) is a gastrointestinal lineage-associated receptor that is retained in several gastrointestinal malignancies, including gastric cancer, making it a biologically attractive ADC target (55). Nevertheless, the clinical experience with GCC-directed ADCs illustrates the difficulty of translating target expression into therapeutic benefit. TAK-164 showed preclinical activity but encountered a narrow therapeutic window in a first-in-human study, with hepatotoxicity limiting dose escalation and preventing achievement of an exposure considered sufficient for meaningful clinical activity (56). The earlier MMAE-based ADC TAK-264 similarly demonstrated only modest activity in GCC-positive gastric and gastroesophageal junction cancer, and subsequent phase II development was discontinued because of limited efficacy (57, 58). These experiences suggest that target prevalence alone is insufficient for ADC success and that payload potency, therapeutic index, tumor penetration, and target accessibility must be considered together when developing GCC-directed strategies.

### TROP2

TROP2 is a tumor-associated transmembrane glycoprotein that is frequently overexpressed across epithelial malignancies and has emerged as an important ADC target because of its high surface abundance and efficient payload delivery (59, 60). Although clinical evidence in gastric cancer remains limited, several TROP2-directed ADCs are under investigation. SKB264 (sacituzumab tirumotecan), which carries a topoisomerase I inhibitor payload, has demonstrated potent preclinical activity and early clinical signals across advanced solid tumors (61, 62). Sacituzumab govitecan has also shown antitumor activity in gastric cancer models, while acquired resistance to its SN-38 payload can involve ABCG2-mediated drug efflux, providing a mechanistic example of payload-specific ADC resistance (63, 64). Datopotamab deruxtecan is also being explored in advanced gastric cancer (65). Thus, TROP2 represents a promising emerging target, but its ultimate role in gastric cancer will depend on prospective clinical validation and improved understanding of target expression and payload resistance.

### Nectin-4

Nectin-4 is a cell-adhesion molecule that is aberrantly expressed in several solid tumors. In gastric cancer, increased Nectin-4 expression has been associated with tumor progression and unfavorable prognosis, supporting its potential as a therapeutic target (66, 67). Although clinical development of Nectin-4-directed ADCs has been led primarily by other tumor types, gastric cancer-specific preclinical evidence is emerging. A Nectin-4-directed nanobody–drug conjugate demonstrated potent antitumor activity in gastric cancer models, supporting the feasibility of this target in GC (68).

Nectin-4 may be particularly relevant in the context of acquired resistance to HER2-directed ADCs. Recent studies indicate that drug-tolerant persisting tumor cells generated after HER2-directed ADC exposure can lose HER2 while acquiring increased Nectin-4 expression, creating a potential therapeutic vulnerability to Nectin-4-directed treatment (69). This adaptive target switching provides a biological rationale for sequential or dual-target ADC strategies and illustrates how clonal evolution during therapy may reshape the actionable antigen landscape in gastric cancer.

### EGFR

EGFR is frequently dysregulated in gastric and gastroesophageal junction cancers, although previous attempts to therapeutically exploit EGFR signaling have produced inconsistent clinical benefit, underscoring the need for improved biomarker selection and drug-delivery strategies (70). MRG003, an EGFR-directed ADC carrying an MMAE payload, has entered clinical evaluation in advanced solid tumors and is being investigated specifically in EGFR-positive, HER2-negative advanced gastric cancer (71, 72). In parallel, bispecific ADC strategies may provide a means of addressing receptor heterogeneity: BL-B01D1 targets both EGFR and HER3 and is currently being evaluated in gastrointestinal malignancies, including gastric cancer, alone and in combination-based approaches (73). These strategies suggest that the future value of EGFR-directed ADC therapy may depend less on EGFR expression alone and more on biomarker refinement and multi-target approaches capable of overcoming intratumoral heterogeneity.

### Mucin 1

MUC1 is an epithelial mucin that is aberrantly expressed and glycosylated in multiple malignancies, including gastric cancer, where altered expression has been associated with tumor progression and adverse clinical features (74). Its extracellular accessibility and tumor-associated glycoforms have prompted the development of ADCs targeting either MUC1-associated glycans or the MUC1 protein backbone (75). Several MUC1-directed ADCs have demonstrated preclinical antitumor activity, including constructs targeting the CA6 glycoepitope or MUC1-C (76, 77). More recently, DS-3939a, an ADC directed against tumor-associated MUC1, has entered first-in-human clinical evaluation in advanced solid tumors (78). Although clinical evidence in gastric cancer remains preliminary, MUC1 illustrates the potential of exploiting tumor-associated glycosylation and cancer-specific antigen conformations to expand the ADC target repertoire beyond conventional receptor tyrosine kinases.

### B7-H3

B7-H3 (CD276) is an immune-regulatory transmembrane molecule that has emerged as an attractive therapeutic target because of its tumor-associated expression and its potential role in immune evasion. In gastric cancer, B7-H3 expression has been linked to aggressive tumor biology, and co-expression of B7-H3 and HER2 was associated with poorer survival, suggesting that B7-H3 may define a biologically and immunologically relevant tumor subset. Several B7-H3-directed ADCs, including YL201 and ifinatamab deruxtecan (I-DXd), have entered early-phase clinical development in advanced solid tumors. YL201, a B7-H3-directed ADC carrying a topoisomerase I inhibitor payload, demonstrated encouraging antitumor activity in a multicenter phase 1/1b study across multiple refractory solid tumors. However, gastric cancer-specific clinical efficacy remains insufficiently established, and B7-H3 should therefore currently be considered an emerging rather than clinically validated ADC target in GC. Its dual relevance as a tumor-associated antigen and an immune-regulatory molecule makes B7-H3 particularly attractive for future ADC development and immunotherapy-oriented combination strategies (79, 80).

## Resistance mechanisms to ADCs in gastric cancer

Resistance to ADC therapy in gastric cancer is multifactorial and can arise at several stages of the ADC pharmacological pathway. A major mechanism is heterogeneous or reduced target-antigen expression. Gastric cancer is characterized by substantial intratumoral and interlesional heterogeneity, particularly for HER2. In the GATSBY biomarker analysis, T-DM1 activity was greater in tumors with higher HER2 expression, suggesting that heterogeneous or insufficient antigen density may limit effective ADC delivery (37). Under therapeutic pressure, antigen-positive clones may be preferentially eliminated, allowing antigen-low or antigen-negative subclones to expand.

Resistance may also occur through altered ADC internalization and intracellular trafficking. Following antigen binding, ADCs require efficient endocytosis, endosomal–lysosomal trafficking, and payload release. Alterations in these processes, including caveolae-mediated internalization, impaired lysosomal proteolysis, and reduced function of lysosomal transporters such as SLC46A3, can decrease intracellular exposure to active payloads and contribute to resistance (81–83).

A third mechanism involves payload-specific resistance. For example, resistance to the SN-38 payload of sacituzumab govitecan has been associated with increased ABCG2-mediated drug efflux in gastric cancer models (64). These findings indicate that resistance may occur independently of target-antigen expression when tumor cells acquire mechanisms that reduce intracellular payload accumulation or sensitivity.

Importantly, ADC treatment may also induce dynamic target switching and tumor evolution. Drug-tolerant persisting cells emerging after HER2-directed ADC exposure may lose HER2 while acquiring increased Nectin-4 expression, creating potential sensitivity to alternative target-directed therapy (69). Spatial profiling of patient-matched HER2-positive gastric cancers has further revealed heterogeneous resistance programs following HER2-directed therapy, including epithelial–mesenchymal transition, altered immune-related signaling, increased CLDN18.2 expression after trastuzumab resistance, and HLA loss and metabolic reprogramming in T-DXd-resistant tumors (84). These observations suggest that ADC resistance should be regarded as an evolving process involving target plasticity, intracellular drug-processing defects, payload resistance, and changes in the tumor microenvironment rather than as a single static mechanism.

For patients with HER2-positive gastric cancer who progress on T-DXd, subsequent targeted treatment should ideally be guided by reassessment of tumor-antigen expression rather than by the baseline HER2 status alone. Loss or spatial heterogeneity of HER2 may emerge under therapeutic pressure; therefore, repeat biopsy or molecular reassessment of a progressing lesion may help determine whether HER2 remains an actionable target. If HER2 expression is retained, switching to a HER2-directed ADC with a different epitope, linker, or payload represents a biologically rational strategy. In preclinical breast and gastric cancer models, RC48, which recognizes a different HER2 epitope and carries an MMAE payload, retained activity in tumors resistant to T-DM1 or T-DXd, although clinical efficacy specifically after T-DXd progression remains to be established (85). If HER2 expression is lost or substantially reduced, reassessment of alternative surface targets such as CLDN18.2, Nectin-4, TROP2, HER3, or B7-H3 may provide opportunities for enrollment in biomarker-selected clinical trials. In particular, HER2-directed ADC exposure has been associated with HER2 loss and increased Nectin-4 expression in drug-tolerant tumor cells, supporting the concept of treatment-induced target switching (69). Bispecific ADCs, sequential ADCs with different payload classes, and dual-ADC strategies may also help overcome heterogeneous or evolving target expression, but these approaches remain investigational. Thus, post-T-DXd therapy should increasingly be viewed as a biomarker-adaptive strategy based on longitudinal reassessment of both target expression and resistance mechanisms.

Collectively, ADC resistance in gastric cancer should be viewed as a dynamic and multifactorial process involving changes in target-antigen expression, intracellular trafficking and payload release, payload-specific resistance, and treatment-induced molecular and immune evolution. These mechanisms may coexist within the same tumor and evolve during treatment, supporting longitudinal reassessment of target expression and resistance biology as well as the development of sequential, alternative-target, bispecific, and dual-ADC strategies. The major mechanisms of ADC resistance relevant to gastric cancer, together with representative evidence and potential therapeutic implications, are summarized in **Table 3**.

**Table 3:** Major mechanisms of resistance to ADCs relevant to gastric cancer.

Resistance mechanism	Biological basis	Representative evidence relevant to GC	Therapeutic implication
Target heterogeneity or loss	Coexistence and selection of antigen-high, antigen-low, and antigen-negative clones	Higher HER2 expression was associated with greater T-DM1 activity in GATSBY biomarker analysis (37)	Re-biopsy, quantitative target assessment, bystander-capable ADCs
Altered internalization	Changes in endocytic pathways reduce productive ADC trafficking	Caveolae-mediated internalization has been associated with T-DM1 resistance (81)	Optimize internalization and trafficking properties
Impaired lysosomal processing	Reduced lysosomal proteolysis decreases payload release	Reduced lysosomal proteolysis and altered SLC46A3 function can impair T-DM1 processing (82, 83)	Linker/payload optimization
Payload resistance/drug efflux	Increased efflux or reduced payload sensitivity	ABCG2-mediated resistance to SN-38-containing ADCs demonstrated in gastric cancer models (64)	Alternative payloads or payload-class switching
Treatment-induced target switching	Therapy selects tumor cells with altered antigen profiles	HER2 loss with increased Nectin-4 expression after HER2-directed ADC exposure (69)	Sequential or dual-target ADC strategies
Adaptive molecular/immune evolution	EMT, immune escape, metabolic adaptation	HLA loss, metabolic reprogramming, immune-related changes in T-DXd-resistant GC (84)	Combination therapy and longitudinal molecular profiling

## Biological determinants of ADC response and progression in gastric cancer

### Immune-hot versus immune-cold gastric cancer

Gastric cancer exhibits marked heterogeneity in its tumor immune microenvironment and can broadly range from immune-inflamed (“hot”) to immune-excluded or immune-desert (“cold”) phenotypes. Immune-hot tumors are generally characterized by abundant tumor-infiltrating lymphocytes, particularly CD8^+^ T cells, active interferon-related signaling, enhanced antigen presentation, and frequent expression of immune-checkpoint molecules such as PD-L1. MSI-high and Epstein–Barr virus-positive gastric cancers are enriched for these immune-inflamed features, although substantial heterogeneity exists even within these molecular subtypes. In contrast, immune-cold tumors contain few functional effector T cells or exhibit T-cell exclusion, and may be dominated by stromal barriers, regulatory T cells, M2-polarized macrophages, myeloid-derived suppressor cells, or other immunosuppressive components. Importantly, an immune-hot phenotype does not necessarily indicate effective antitumor immunity, because infiltrating T cells may be functionally exhausted or restrained by inhibitory checkpoint signaling (86).

This distinction has direct implications for ADC therapy. In an immune-cold tumor, direct cytotoxicity alone may achieve initial tumor reduction without generating durable systemic immunity. Conversely, selected ADC payloads can induce immunogenic tumor-cell death, resulting in tumor-antigen release, dendritic-cell activation, enhanced antigen presentation, and recruitment of effector T cells (29–31). ADC treatment may therefore shift some tumors toward a more inflamed immune state. However, this immune activation can simultaneously induce adaptive checkpoint signaling, including PD-L1 upregulation, thereby limiting the durability of the immune response. This biological sequence provides a mechanistic rationale for combining ADCs with PD-1/PD-L1 blockade: ADCs may provide tumor debulking and immune priming, whereas immune checkpoint inhibitors can release the adaptive inhibitory signals that emerge after treatment.

### Biological and prognostic differences between ADC target-positive and target-negative tumors

ADC target expression may have biological and prognostic significance beyond its role as a predictive biomarker for drug binding. However, the relationship between target positivity and prognosis is target-specific rather than universal. HER2-positive gastric cancer represents a distinct molecular and immune subset, with differences in PD-L1 expression and immune-cell composition compared with HER2-negative disease (33, 34). A recent pooled analysis also suggested that HER2 overexpression is associated with poorer overall survival in both resectable and advanced gastric cancer (87), although this relationship may be modified by the availability of HER2-directed therapies.

CLDN18.2-positive tumors also appear to represent a biologically distinct subgroup, although the prognostic impact of CLDN18.2 expression alone remains inconsistent across studies. CLDN18.2 positivity has been associated with diffuse-type histology, altered PD-L1 expression, and distinct T-cell and stromal infiltration patterns. Importantly, CLDN18.2 expression is dynamic rather than fixed. Serial-biopsy analyses have shown that CLDN18.2 can be lost or gained during chemo-immunotherapy, indicating that a tumor classified as target-positive at baseline may not retain the same target status during disease evolution (88).

Similarly, high HER3 and Nectin-4 expression has been associated with adverse clinicopathological features or poorer survival in gastric cancer (44, 66, 67, 89, 90). Collectively, these findings indicate that ADC target expression should not be viewed solely as a binary companion-diagnostic variable. Rather, target-positive and target-negative tumors may differ in lineage, signaling dependencies, immune context, and evolutionary behavior. Future ADC trials should therefore integrate quantitative target expression with immune biomarkers and longitudinal reassessment of target status.

### Spatial transcriptomics and spatial mapping of ADC target expression

Conventional biomarker assessment using immunohistochemistry or *in situ* hybridization provides essential information for ADC target selection but may incompletely capture the pronounced spatial heterogeneity of gastric cancer, particularly when evaluation is based on limited biopsy material. Spatial transcriptomics preserves the anatomical context of gene expression and enables simultaneous characterization of target-positive, target-low, and target-negative tumor regions together with their surrounding immune and stromal niches. This approach is particularly relevant to HER2-directed ADC therapy. Recent spatial single-cell multi-omics profiling of gastric cancer demonstrated extensive spatial heterogeneity across different levels of HER2 expression and revealed distinct immune and stromal microenvironments associated with HER2-high, intermediate, low, and negative tumors. Notably, spatial HER2 heterogeneity was associated with less favorable therapeutic responses and poorer outcomes, supporting the concept that average target expression alone may not adequately predict sensitivity to HER2-directed treatment (91).

### Spatially resolved profiling may also provide insight into treatment-induced target evolution and mechanisms of ADC resistance.

In patient-matched HER2-positive gastric cancers analyzed before and after HER2-directed therapy, spatial transcriptomic profiling identified heterogeneous resistance programs, including epithelial–mesenchymal transition, alterations in immune-related signaling, increased CLDN18.2 expression after trastuzumab resistance, and HLA loss and metabolic reprogramming in T-DXd-resistant tumors (84). These findings illustrate how spatial profiling may reveal alternative therapeutic targets that emerge under treatment pressure and thereby inform sequential, bispecific, or dual-ADC strategies. Spatial heterogeneity is also relevant to CLDN18.2: substantial intratumoral heterogeneity and discordance between primary and metastatic lesions have been reported in metastatic gastric cancer (92). Accordingly, spatial transcriptomics, particularly when integrated with multiplex immunofluorescence, spatial proteomics, and conventional immunohistochemistry, could refine ADC patient selection by defining not only whether a target is present, but also where it is expressed, how uniformly it is distributed, and which immune or stromal niches coexist with target-positive tumor cells. Importantly, because ADC binding ultimately depends on cell-surface protein abundance and accessibility, transcriptomic target expression should complement rather than replace validated protein-level assays.

## Combination therapy of ADC in GC

Targeted therapy has transformed the management of many malignancies, yet only a minority of patients derive durable benefit, and long-term survival in advanced GC remains unsatisfactory (93). A strong biological rationale now supports combining ADCs with other modalities to overcome tumor heterogeneity, delay or circumvent resistance, and enhance therapeutic efficacy (94, 95). In this context, ADC-based combinations with chemotherapy, molecularly targeted agents, radiotherapy, endocrine therapy, and immunotherapy are being actively explored in preclinical models and clinical trials, with the aim of achieving synergistic—not merely additive—anticancer effects and extending survival beyond that achievable with monotherapy (95). The following section summarizes representative ADC-based combination strategies in GC, including combinations with chemotherapy, monoclonal antibodies, immune checkpoint inhibitors, and emerging bispecific or dual-ADC approaches.

### ADCs combined with chemotherapy

Many conventional cytotoxic agents, including antimetabolites, platinum compounds, and topoisomerase inhibitors, exert their antitumor activity by inducing DNA damage and perturbing the cell cycle, particularly through S-phase targeting and G2/M arrest (96). In HER2-positive advanced GC, anti-HER2 targeted therapy combined with chemotherapy is the current standard first-line approach, and ADC–chemotherapy combinations are being actively explored. However, combining ADCs with cytotoxic agents can be constrained by overlapping toxicities: in the DESTINY-Gastric03 trial, T-DXd plus 5-fluorouracil or capecitabine in metastatic HER2-positive GC led to dose-limiting stomatitis and a high incidence of grade ≥3 adverse events, underscoring the need to balance efficacy with tolerability when designing ADC–chemotherapy regimens (97).

### ADCs combined with monoclonal antibody therapy

Binding-site barriers (BSBs) arise when high-affinity monoclonal antibodies bind densely to antigens around tumor vasculature, become sequestered, and fail to penetrate into regions distant from blood vessels, leading to heterogeneous intratumor distribution and suboptimal efficacy (98). In GC models, recent work in the MKN74 cell line has shown that ADCs used together with their parental antibodies can partially circumvent BSBs and improve penetration into tumor tissue (99).

More broadly, BSBs in HER2-positive GC reflect the interplay of high antigen density, rapid antigen internalization, and slow interstitial diffusion, all of which limit deep antibody penetration into solid tumors (98). In the HER2-positive NCI-N87 GC model, combined blockade with T-DM1 and pertuzumab produced greater tumor regression than either agent alone, with suppression of EGFR and HER3 phosphorylation, downregulation of AKT signaling, increased caspase-3/7 activity, and enhanced ADCC (100). Strategies such as transient competitive inhibition using carrier antibodies or single-domain antibodies have further improved intratumor distribution: co-administration of an anti-trastuzumab single-domain antibody (1HE) increased trastuzumab penetration from the vasculature and expanded the trastuzumab-positive tumor area, and when combined with a single dose of T-DM1, significantly improved survival in NCI-N87 xenografts (101). Collectively, these preclinical data indicate that rational monoclonal antibody–ADC combinations can mitigate binding-site barrier-related limitations, deepen target inhibition, and enhance antitumor activity; however, clinical validation in gastric cancer remains necessary.

### ADC–ICI combinations: clinical translation

As discussed above, ADC-mediated immunogenic tumor-cell death, antigen presentation, and adaptive checkpoint upregulation provide a mechanistic basis for combining ADCs with immune checkpoint blockade.

Preclinical evidence supports this concept: HER2-targeted ADCs such as XMT-1522 and T-DXd have demonstrated enhanced antitumor activity when combined with PD-1/PD-L1 blockade (31, 32). In GC-specific models, MRG002 combined with anti-PD-1 further augmented tumor regression (42). Clinically, RC48 plus toripalimab (JS001) achieved an ORR of 43%, a disease control rate of 75%, a median PFS of 6.2 months, and a median OS of 16.8 months in patients with HER2-expressing locally advanced or metastatic GC (102). T-DXd has been shown to increase tumor-infiltrating CD8^+^ T cells and upregulate PD-L1 and MHC class I expression (31), and is now being evaluated alone and in combination with the PD-L1 antibody durvalumab in gastric or gastroesophageal junction cancer (NCT04379596) (103). Collectively, these findings support continued investigation of ADC–ICI combinations as a strategy to enhance immune responsiveness in biomarker-defined subsets of advanced GC.

### Bispecific and dual-ADC strategies

Bispecific ADCs and dual-ADC combinations represent conceptually distinct strategies for overcoming intratumoral antigen heterogeneity and treatment-induced target escape. Bispecific ADCs recognize two antigens or epitopes within a single construct, potentially broadening tumor-cell coverage and reducing escape of antigen-low subclones. BL-B01D1 is a first-in-class EGFR–HER3 bispecific ADC that has demonstrated preliminary antitumor activity in a first-in-human phase 1 study of advanced solid tumors (104).

Dual-ADC therapy, in contrast, involves the concurrent or sequential use of two ADCs. Even when two ADCs target the same antigen, differences in epitope recognition, payload class, internalization, and bystander activity may provide complementary antitumor effects. Recent preclinical findings support this strategy in gastric cancer. RC48 retained activity in breast and gastric cancer models resistant to T-DM1 or T-DXd, and combinations of RC48 with either T-DM1 or T-DXd produced greater antitumor activity than the corresponding single agents (85). These findings suggest that combining HER2-directed ADCs with distinct epitopes and payloads may help overcome resistance and intratumoral heterogeneity.

A complementary dual-target approach is to combine ADCs directed against different surface antigens. In HER2- and Nectin-4-coexpressing gastric cancer models, the combination of the HER2-directed ADC T-DM1 with the Nectin-4-directed ADC enfortumab vedotin produced greater antitumor activity than either ADC alone, including in resistant xenograft models (105). Such findings provide a rationale for dual-target ADC combinations that broaden clonal coverage and exploit treatment-induced target switching. However, these strategies remain preclinical, and potential challenges including overlapping toxicity, optimal dosing, and treatment sequencing require further investigation before clinical translation.

## Limitations of ADCs

Despite their clinical activity, ADCs remain subject to several biological, pharmacological, and clinical limitations. Tumor resistance is a major challenge and may arise through heterogeneous or reduced target-antigen expression, altered internalization and intracellular trafficking, impaired lysosomal processing and payload release, payload-specific resistance, drug efflux, and treatment-induced target switching. These mechanisms are discussed in detail above and highlight the dynamic nature of ADC resistance in gastric cancer. In addition, spatial and temporal heterogeneity of target expression may limit patient selection based on a single biopsy and can lead to incomplete tumor-cell coverage during treatment.

Off-target toxicity represents another important limitation. Premature linker cleavage or deconjugation in the circulation can result in early release of the cytotoxic payload, increasing systemic exposure and damage to non-tumor tissues. Conversely, even when linker stability is adequate, on-target/off-tumor toxicity may occur when the target antigen is expressed at low levels in normal tissues. The bystander effect may also be double-edged: although membrane-permeable payloads can improve killing of neighboring antigen-low tumor cells, diffusion beyond the intended tumor compartment may increase toxicity. ADC pharmacokinetics and therapeutic index are further influenced by DAR, hydrophobicity, aggregation, linker stability, payload properties, and systemic clearance. Finally, overlapping toxicities may constrain ADC-based combination regimens, while incomplete biomarker standardization and the need for longitudinal reassessment of target expression remain important barriers to optimal patient selection. Collectively, these limitations underscore the need for improved linker stability, optimized ADC architecture, more precise biomarker strategies, and rational sequencing or combination approaches to maximize efficacy while minimizing toxicity (106).

## Conclusions

Antibody–drug conjugates have evolved from an experimental therapeutic concept into a clinically validated treatment modality that is reshaping precision therapy for gastric cancer. HER2-directed ADCs and emerging ADC strategies targeting CLDN18.2 and other tumor-associated antigens have broadened therapeutic opportunities, although their clinical efficacy remains constrained by tumor heterogeneity, acquired resistance, imperfect biomarker selection, and treatment-related toxicities. Future development will require continued optimization of antibody engineering, linker–payload design, target selection, and rational combination strategies.

Importantly, ADCs should not be regarded solely as vehicles for selective cytotoxic drug delivery. Increasing evidence indicates that their therapeutic activity can intersect with antitumor immunity through Fc-dependent immune-effector functions, immunogenic tumor-cell death, enhanced antigen presentation, recruitment of effector T cells, and remodeling of the tumor immune microenvironment. These immunological effects provide a mechanistic rationale for combining ADCs with immune checkpoint blockade and may contribute to heterogeneous therapeutic responses among patients with gastric cancer. Future studies should therefore integrate tumor-antigen expression with immune-contextual biomarkers and longitudinal characterization of the tumor microenvironment to better identify patients most likely to benefit from ADC-based therapies. Bridging targeted cytotoxicity with immune modulation may represent an important direction for the next generation of ADC-based treatment in gastric cancer.
